# The Influence of Furfuryl Resin Type—Classical and Designed for Sand 3D Printing—On Cast Iron Casting Microstructure and Surface Roughness

**DOI:** 10.3390/polym17212920

**Published:** 2025-10-31

**Authors:** Katarzyna Major-Gabryś, Dawid Halejcio, Andrzej Fijołek, Jan Marosz, Marcin Górny

**Affiliations:** Faculty of Foundry Engineering, AGH University of Krakow, Mickiewicza 30, 30-059 Krakow, Polandmarosz@agh.edu.pl (J.M.); mgorny@agh.edu.pl (M.G.)

**Keywords:** furfuryl resin, molding sands, 3D printing, cast iron

## Abstract

Resin-based binders are one of the main materials used in foundry molding and core sands. Self-curing sand with furfuryl resin is one of the most popular technologies in the production of molds and cores for complex, critical castings made of iron and non-ferrous alloys. It has dominated small-batch production and the production of large-sized castings. This work is part of the research on new molding sands for mold additive manufacturing (3D printing). Three-dimensional printing technology in the production of sand-casting molds and cores is finding increasing industrial application in the production of castings from non-ferrous metal alloys. The aim of the research presented in this paper was to determine the influence of furfuryl resin type (classical and designed for 3D printing of sand molds) on cast iron casting properties. The pouring parameters were elaborated on the basis of the MAGMA software. Microscopic observations of castings, produced in classical and 3D-printed molds, were conducted, as well as an assessment of the roughness of the samples. The gas emissions from molding sands with both types of furfuryl resin were tested and analyzed in the context of the roughness of the castings obtained. It was proven that molding sand with furfuryl resin designed for 3D printing was characterized by lower gas emissions, which, in the case of molding sands with organic binders, is beneficial from an environmental point of view.

## 1. Introduction

Resin-based binders are one of the main materials used in foundry molding and core sands. This work is part of the research on the influence of binders used in the production of casting molds and cores on the surface quality of castings. Furfuryl resins and resins modified with furfuryl alcohol used as foundry binders contain 7–90% (practically 30–80%) furfuryl alcohol. The addition of alcohol accelerates the hardening process and increases the bonding capacity, which makes it possible to reduce the addition of binder to the molding sand. Furfuryl resins undergo spatial cross-linking under the influence of heating or chemical agents.

Furfuryl resins used for the preparation of self-curing molding sands are obtained through polycondensation of furfuryl aldehyde (furfural C_4_H_3_O·CHO) with phenol or ketones (especially acetone) or polycondensation of furfuryl alcohol (C_4_H_3_O·CH_2_OH) with formaldehyde, urea, phenol, or other compounds. Furfuryl resins undergo spatial cross-linking under the influence of elevated temperatures or chemical agents. In an acidic environment, furfuryl alcohol condenses with heat release, turning into a solid but brittle substance. For this reason, plasticized resins containing furfuryl alcohol are used in combination with other resin components, primarily urea–formaldehyde and resole resins [1,2,3]. Furfuryl resins and resins modified with furfuryl alcohol can contain from 7 to over 90% furfuryl alcohol [1]. Usually, this additive ranges from 30 to 85% according to [1] or from 50 to 95% according to [4].

The higher the furfuryl alcohol content, the greater the binding capacity and the faster the resin curing process [1]. An equally important parameter is the nitrogen content in furfuryl resin, which can range from 0 to 11%. Furfuryl resins with low nitrogen content (below 1%) and low water content are used in steel and aluminum castings [5]. In an acidic environment, furfuryl resin condenses with heat release. Therefore, organic and inorganic acids, usually a mixture of both, are used as hardeners for self-curing powder with furfuryl resin. Organic sulfonic acids, as well as inorganic sulfuric or phosphoric acids, are used. Aromatic sulfonic acids are used in most hardeners, sometimes in a mixture with orthophosphoric acid [1]. Nitric acid (HNO_3_) is not used due to the accumulation of nitrogen in the reclaim. Similarly, hydrochloric acid (HCl) forms harmful compounds when combined with hydrocarbons at high temperatures. Carbonic acid (H_2_CO_3_) and others, such as formic acid (HCOOH) and acetic acid (CH_3_COOH), are too weak and delay the curing process [1,2].

The technology of self-curing molding and core sands with furfuryl resins ensures the procurement of castings with high dimensional accuracy, the ability to prepare complex molds, bonding at ambient temperatures, an easy mold-making process, good knockout properties, and the ability to mechanically regenerate the used molding sands. The disadvantages of this technology include, first of all, the high harmfulness of the gases emitted, mainly during pouring with liquid alloy, and the problem with the disposal of post-regeneration waste from the used molding sands.

**Figure 1:**
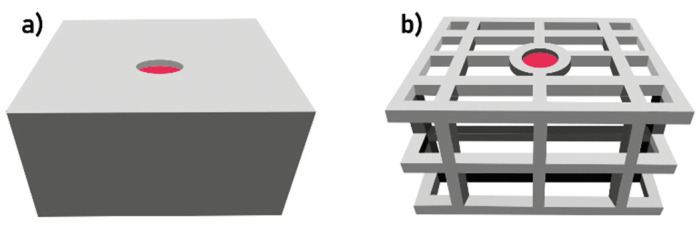
Printed molds: (**a**) classic type of mold; (**b**) mold with cavities (shell–truss mold) [10].

**Figure 2:**
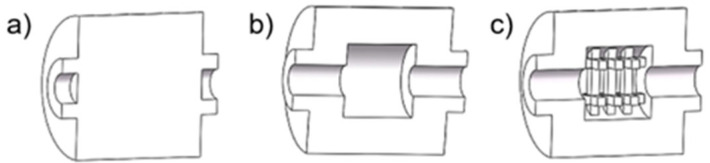
Scheme of cores: (**a**) solid; (**b**) with a cavity; (**c**) with a truss structure [14].

Additive manufacturing (AM) techniques have become part of Industry 4.0 and one of the methods of rapid prototyping. It is believed that in the future, they may partially replace traditional methods of mold and core production. Among additive methods, 3D printing is classified as one of those methods that do not require additional processing after the printing process is completed [6,7,8,9]. This technique allows for a very good surface quality of castings, which has a decisive impact on the quality of cast components. From the point of view of molding technology, the use of 3D printing in the production of sand molds and cores allows for the introduction of solutions that are impossible to achieve using traditional methods [10,11,12,13]. Examples are shown in Figure 1 (printed shell–truss mold and traditional mold: saving two-thirds of the molding sand and reducing the cooling time by 25% compared to the traditional mold type [10]) and in Figure 2 (a core with a cavity [14] was printed for better heat dissipation, and a core with a truss structure was created). The authors of [14] proved that when pouring cast steel into a mold, the hollow sand core has lower expandability than a solid core. This reduces the likelihood of cracks forming on the inner wall of the casting.

In 3D printing technology, quartz sand [7,8] is mainly used as the matrix, with a grain size ranging from 0.14 to 0.25 mm [15,16], forming successive layers with a thickness of 0.28 to 0.50 mm [17]. As binders, two resins are mainly used: phenolic resin, added to the molding sand at 2.2–2.4% [11], and most often furfuryl resin at 1–3% [11,18], and even 8% according to D. Snelling et al. [19].

**Figure 3:**
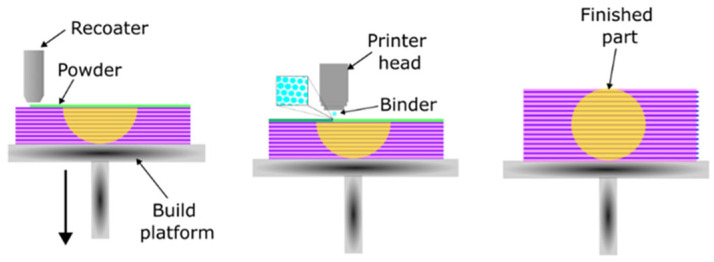
Scheme of the sand 3D printing process using the binder jetting method [17].

In the case of chemical curing printing, the sand matrix is mixed with a curing agent. A device called a recoater applies a layer, which it then levels, and a printhead sprays the binder in a designated area. The process is repeated, applying successive layers until the printed element is completed [19,20,21]. The binder penetrates between the grains, where the curing reaction takes place. Only the part of the molding compound that constitutes the printed element undergoes curing. The mixture of sand and hardener that does not participate in the process can be recycled and used in the production of subsequent elements. A diagram of the 3D printing process for a sand element using binder jetting technology is shown in Figure 3 [17].

**Figure 4:**
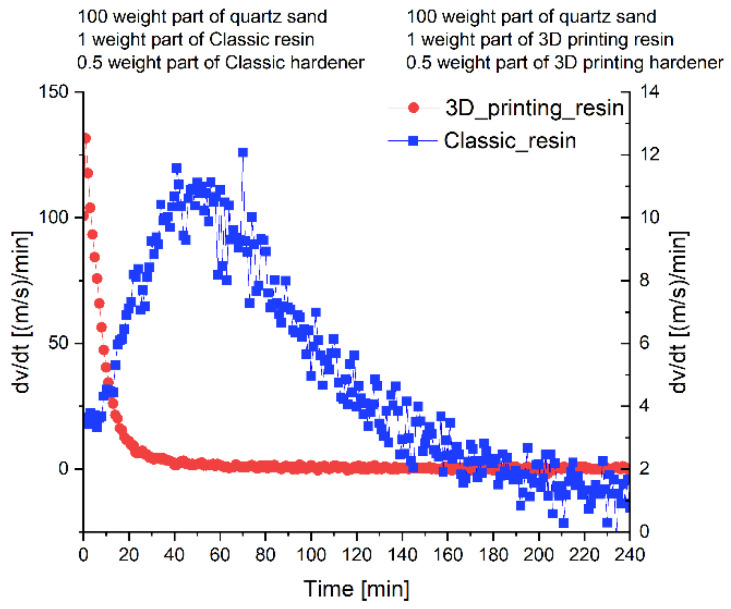
The influence of binder type on the kinetics of molding sands hardening at 20 °C [6].

**Figure 5:**
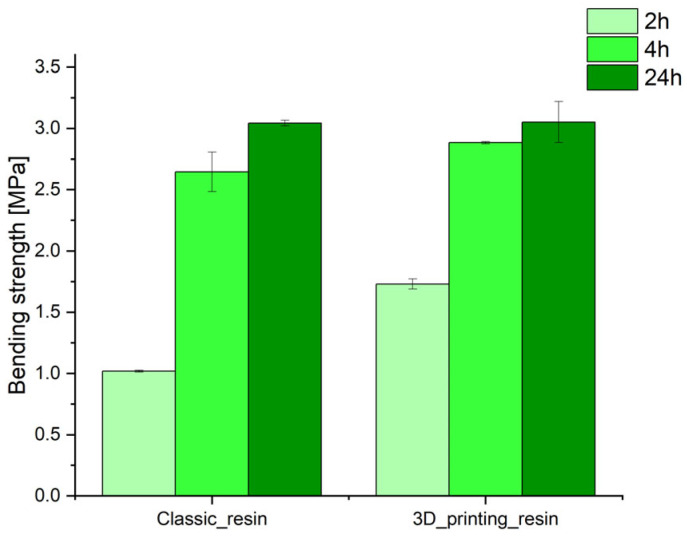
The influence of binder type on selected technological properties of the tested molding sands [6].

From the point of view of preparing molding and core sand, the parameters of the binding materials used are important. Our own research [6] confirmed that furfuryl resin-based binders used for the production of molds and cores in 3D printing technology are characterized by reduced viscosity compared to furfuryl resin-based binders used in traditional mold and core production technology. In addition, it has been shown that the binding system used in 3D printing technology is more reactive, which increases the binding kinetics (Figure 4). A comparison of select properties of molding compounds used for 3D printing and compounds used in traditional technologies is presented in Figure 5 [6].

Three-dimensional printing technology in the production of sand molds and cores is increasingly used in industry for the production of non-ferrous alloy castings. However, this technology is not used in the industrial production of cast iron castings. The newest studies [22,23] on the use of this technology for cast iron casting production have mainly focused on the castings’ quality in terms of the formation of casting defects on their surface. Thus, there is a research gap concerning the impact of mold and core production technology—classical and designed for 3D printing of sand molds—on the formation of the microstructure of gray cast iron. This article presents the results of research on the influence of furfuryl resin type on the quality of castings produced in 3D-printed and traditionally self-cured molds. Within this research, molds for testing the properties of castings were designed, and computer simulations of the metal solidification process were performed. Next, the quality of the castings was tested: the surface quality of the castings and the microstructure, depending on the wall thickness of the casting. Finally, gas emission tests for molding sands with both types of resins were conducted.

## 2. Materials and Methods

### 2.1. Materials

The molds were produced from self-curing molding sands. The following materials were selected as components for molding sands that were subjected to tests:

- Furfuryl resin, with the presence of free formaldehyde in the range of 0.12–0.14%; the amount of furfuryl alcohol was 75% (classic furfuryl resin).
- Hardener—an aqueous solution of paratoluenesulfonic acid (classic hardener).
- Furfuryl resin designed for 3D printing, with furfuryl alcohol content between 70 and 90%.
- A hardener designed for 3D printing, sulfuric acid solution paratoluenesulfonic acid (containing a maximum of 5% H_2_SO_4_).

The compositions of the molding sands are presented in Table 1.

In order to eliminate the influence of grain size distribution on the surface properties of castings, quartz sand with a grain size of 0.20/0.16/0.10, with an average grain size of 0.225 mm; a main fraction share of 96.57%; a density of 1.686 ± 0.027 g/cm^3^; and pH = 6.24 was used in all tests. This matrix is suitable for the production of molds and cores in 3D printing technology.

The main properties of the resin and hardener are presented in Table 2 and Table 3.

### 2.2. Methods

**Figure 6:**
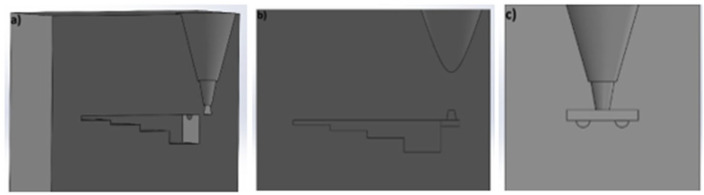
Design of a mold intended for production using 3D printing technology: (**a**) isometric projection of the mold cross-section, (**b**) side view of the mold cavity cross-section, (**c**) cross-sectional view of the sprue system.

Figure 6 shows a 3D-printable mold designed as part of this work. It is a standard mold for a so-called step test. The test consists of making castings with different wall thicknesses (5 mm, 10 mm, 15 mm, and 25 mm) in order to assess the impact of the wall cooling rate on the microstructure of the cast alloy. An additional objective of the research will be to examine the surface roughness of the casting made in the printed mold and to conduct comparative tests of the effect of the type of furfuryl resin on the quality of the castings obtained.

In the first stage of the research, computer simulations of the solidification and cooling process of castings were carried out. The MAGMASOFT 6.1 software was used to perform the simulations. The following simulation parameters were adopted: pouring temperature, 1420 °C; pouring process completed when the mold cavity was completely filled; crystallization process completed when the temperature reached 700 °C. The influence of the type of furfuryl resin used was omitted in the design of the mold pouring process. The influence of this parameter on the cooling and solidification process of the casting is negligible due to the high thermal resistance of the sand mold. These conclusions are confirmed by the literature data [24,25].

A Kocel AJS300 3D printer produced by KOCEL INTELLIGENT MACHINERY LIMITED (Yinchuan, China) was used to produce the mold using 3D printing technology. The matrix with the hardener was mixed in a rotary mixer for 7 min to obtain the optimum sand flowability. The printing parameters were as follows:

- X Print speed: 0.3 m/s;
- Y Print speed: 0.3 m/s;
- Printhead clean after: 4 layers;
- Recoater speed: 100 mm/s;
- Sanding time: 0.6 s;
- Sanding layers: 5;
- Recoater vibration/Blade rotation: 4600 rpm;
- Resolution: 0.06 mm;
- Layer thickness: 0.32 mm.

The printed mold was left to cure at ambient temperature for 24 h. In order to make the mold using traditional technology, the components of the mixture were mixed in a rotary mixer in the following order: matrix + hardener − 60 s, + resin − 60 s. The mold produced with traditional technology was left to cure at ambient temperature for 24 h.

The metal melting was carried out using a medium-frequency induction furnace with a graphite crucible with a 15 kg capacity of charge. The molds were poured with liquid metal with a drain temperature of about 1420 °C and a pouring temperature of about 1410 °C.

Microscopic examinations of casting microstructures were conducted with the use of the Keyence VHX7100 produced by KEYENCE INTERNATIONAL (BELGIUM) NV/SA (Mechelen, Belgium). This microscope is equipped with special software that allows the roughness parameters of the sample to be examined while mapping the surface of the casting.

In the case of organic binders, which burn out during the pouring of liquid casting alloy, a large amount of gas is released from molds and cores. This can lead to casting defects, as the gases cannot escape from the mold quickly enough. On the other hand, the gases escaping from the mold are harmful to the environment, which is also a problem from an ecological point of view. For the above reasons, it is important to use materials with reduced gas emissions. A gas emission test was conducted according to Polish standard BN-76/4024-05 [26]. After reaching a temperature of 1000 °C, a corundum boat with a weighed sample of 2 g is introduced into the quartz tube. The pipe is closed tightly. The other end of the pipe is connected to a peristaltic pump, which is turned on to create negative pressure. The measurement is started, and the quartz tube is moved to the position where the sample is in the heating zone. Placing the sample in the heating zone causes the release of gases that are products of the reactions taking place. This increases the pressure in the system. The pump is automatically turned on to remove the generated gases. The recording of the volume of released gases continues until the pressure stabilizes at the initial value.

## 3. Results

### 3.1. Simulation of the Cooling and Solidification Process of Castings

#### 3.1.1. Simulation of Temperature Distribution During the Casting Process

**Figure 7:**
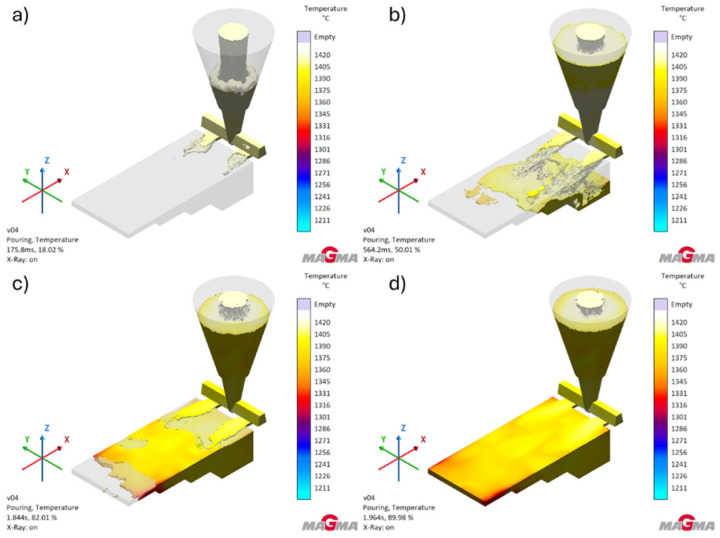
Temperature distribution in the test casting during the pouring process: (**a**) at the moment of filling the gating system (18.00% of simulations); (**b**) at the moment of pouring the casting (50.00% of simulations); (**c**) at the moment of filling the last part of the casting (82.00% of simulations); (**d**) fully filled casting (89.94% of simulations).

Figure 7 shows the temperature distribution during the filling of the cavity of the test casting mold.

An analysis of Figure 7 and the test results obtained shows that the designed gating system ensures correct filling of the mold cavity without significant temperature drops. Slight temperature drops below 1350 °C can only be observed when the mold cavity is completely filled. They occur at the thinnest wall of the casting. The temperature values do not approach the solidus temperature of the alloy and will not have a negative impact on the filling of the mold cavity.

**Figure 8:**
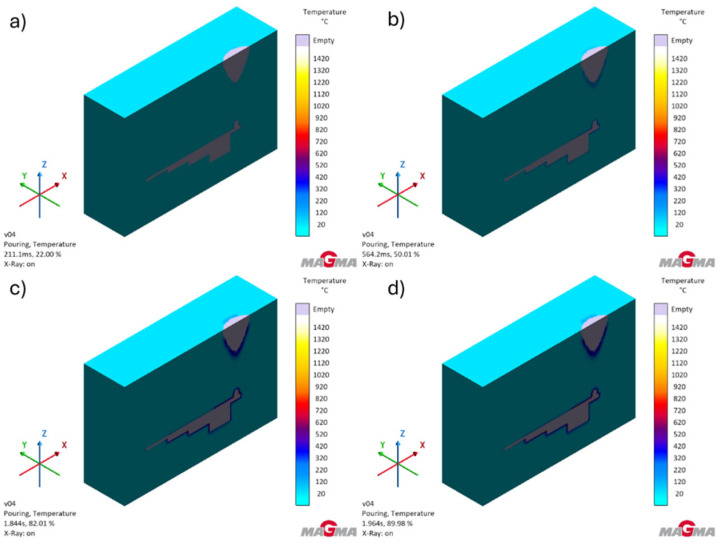
Temperature distribution in the mold during the pouring process: (**a**) after filling the pouring system (22.01% of simulations); (**b**) during casting (54.01% of simulations); (**c**) after pouring the last part of the casting (84.00% of simulations); (**d**) fully filled mold (89.94% of simulations).

Figure 8 shows the temperature distribution in the mold during the pouring process.

The analysis of the heating process shows that the wall thickness of the mold was correctly selected for the tested gray cast iron alloy. The highest temperatures are reached at thermal nodes, i.e., at points where the wall thickness changes.

#### 3.1.2. Simulation of Liquid Metal Flow Velocity During the Pouring Process

**Figure 9:**
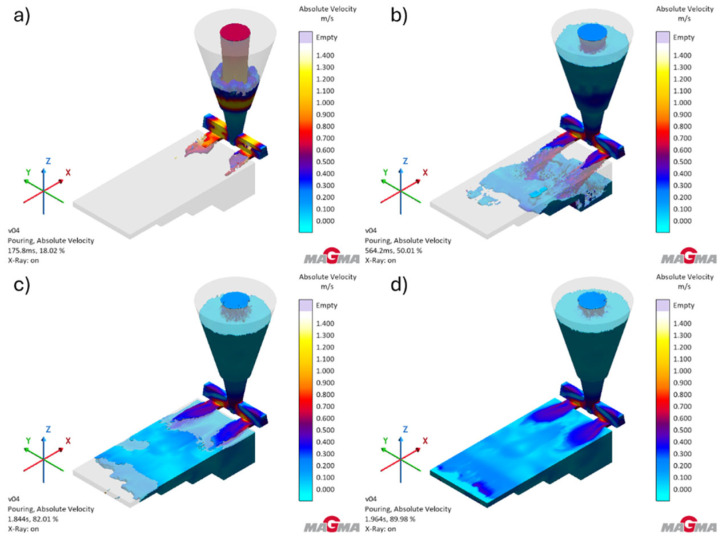
Velocity distribution in the test casting during the pouring process: (**a**) at the moment of filling the gating system (18.02% of simulations); (**b**) at the moment of pouring the casting (50.01% of simulations); (**c**) at the moment of filling the last part of the casting (82.01% of simulations); (**d**) fully filled casting (89.94% of simulations).

Figure 9 shows changes in the flow velocity of liquid metal in the designed casting mold.

Simulations of velocity distribution have shown that molten metal reaches its highest speeds in the sprues. As the mold cavity is filled, the flow becomes more uniform, allowing the mold cavity to be filled smoothly with liquid metal.

#### 3.1.3. Simulation of Solidification and Crystallization—Temperature

**Figure 10:**
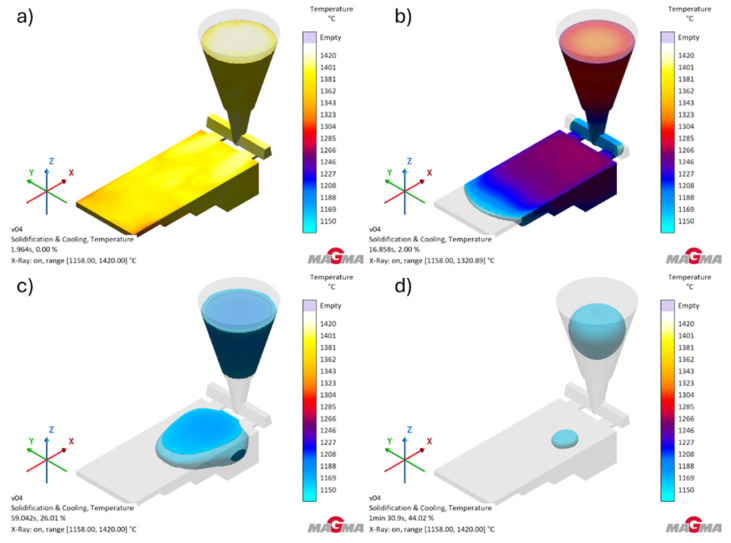
Temperature distribution during solidification and crystallization: (**a**) at the end of the pouring process (0.00% of the simulation); (**b**) at the moment of solidification of the first part of the casting (2.00% of the simulation); (**c**) at the moment of solidification of ¾ of the casting (26.00% of the simulation); (**d**) one step before the solidification of the last part of the casting (44.01% of the simulation).

Figure 10 shows the temperature distribution during the solidification and crystallization of the test casting.

The simulation results showed that the gray cast iron alloy tested in the designed mold very quickly reaches the solidus temperature (1148 °C). This process takes 1 min 56 s. The tests show that the alloy cools completely below the eutectoid transformation temperature (727 °C) after 3 min 43 s. The simulation of the solidification process of the tested alloy shows that the casting is very well fed by the gating system.

#### 3.1.4. Solidification and Crystallization—Probability of Porosity Formation

**Figure 11:**
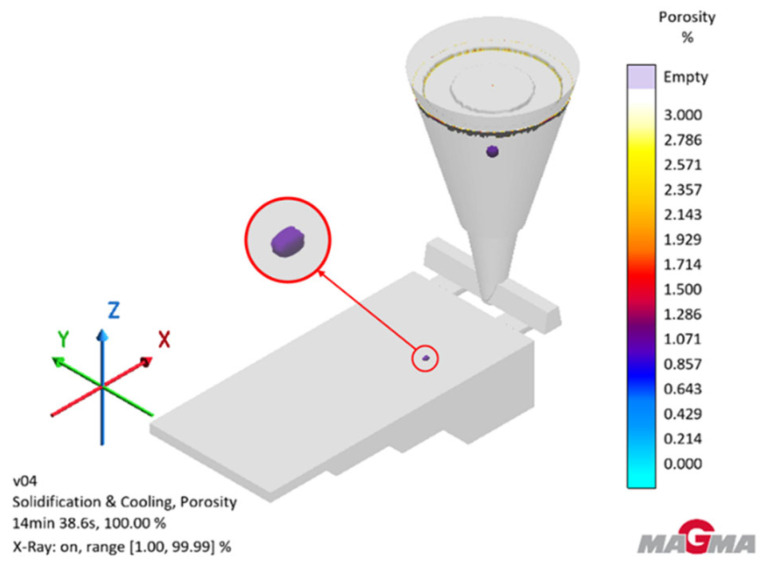
A place where porosity is likely to form during the solidification and crystallization process.

Figure 11 shows simulations of the probability of porosity formation during the solidification and crystallization process.

The simulation shows that there is a low probability of porosity occurring (approximately 1.071%). Porosity occurs in the thickest part of the test casting. The simulation showed that the highest porosity will be found in the sprue, which indicates a well-designed casting mold.

### 3.2. The Microscopic Observations of Casting Microstructures

**Figure 12:**
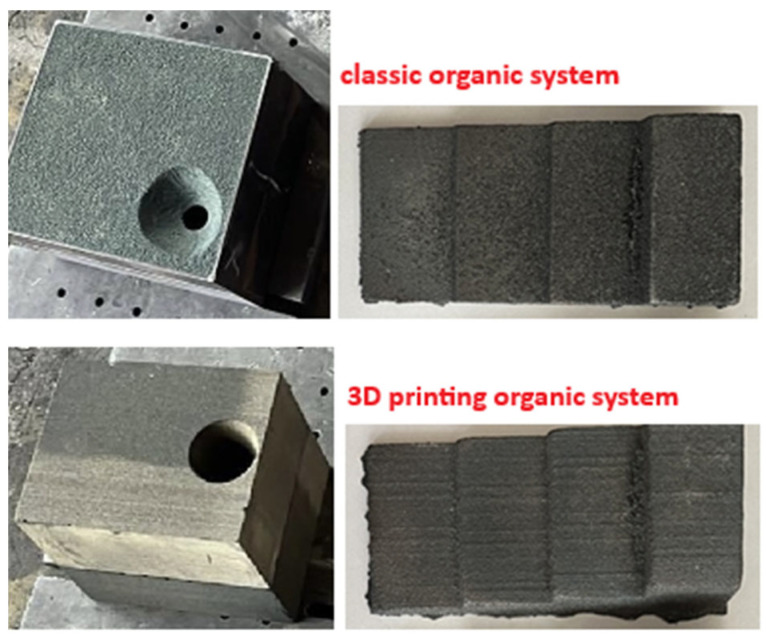
Molds made using classic no-bake technology and 3D printing technology, with castings produced in them.

The molds were poured with gray cast iron with a drain temperature of about 1420 °C and a pouring temperature of about 1410 °C. A chemical composition analysis of the examined alloy is presented in Table 4. Figure 12 shows gray iron castings together with the molds in which they were produced.

**Figure 13:**
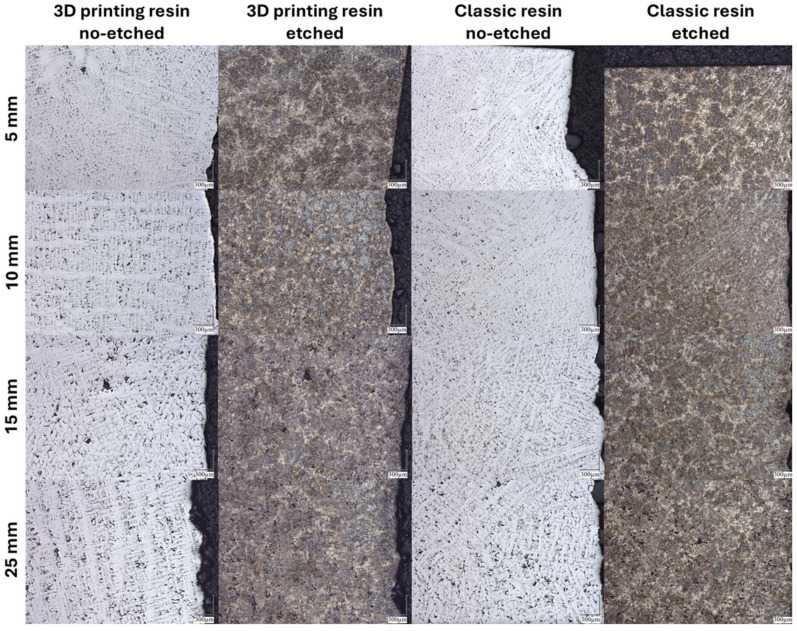
Microstructures of the tested gray cast iron alloys, no-etched and etched, depending on the binder used and the wall thickness of the casting; magnification: 100×.

Figure 13 shows the no-etched and etched microstructures of the tested gray cast iron alloys produced in molds using classic furfuryl resin and resin for 3D printing.

All photos were taken near the wall in direct contact with the molding sand. Metallographic examinations were carried out in accordance with EN ISO 945-1:2008 [27]. Etched samples of the investigated castings revealed local chill zones in thin-walled sections, i.e., 3 mm. The matrix was almost entirely pearlitic, with ferrite occurring at up to 3% within the areas surrounding flake graphite precipitates. In all wall thicknesses of the castings, for both mold types, large dendritic precipitates were observed. No variations in crystallization from the casting wall were detected in the cast iron specimens. The results of the structural analysis of the investigated castings, for both conventional sand molds and 3D-printed molds, are summarized in Table 5.

The secondary dendrite arm spacing parameter and the maximum length of graphite flakes show no significant variation between the two mold types. In contrast, the eutectic grain density per unit area differs considerably, particularly within the thin sections of the step casting. The change in the number of eutectic grains is usually beneficial from the point of view of the formation of type A graphite and indicates good inoculation. In the case of thin-walled castings, there is generally a strong tendency to form type D graphite (Table 5), which is associated with high cooling rates. Despite the difference in the number of eutectic grains in thin-walled castings (classic resin and 3D printing resin), this did not affect the type of graphite formed. The effect of the higher number of eutectic grains in the classic resin can be attributed to the influence of both molding materials on slightly different solidification conditions of the graphite eutectic, i.e., on the number of graphite and cementite eutectic nuclei formed.

### 3.3. The Surface Roughness of Castings

**Figure 14:**
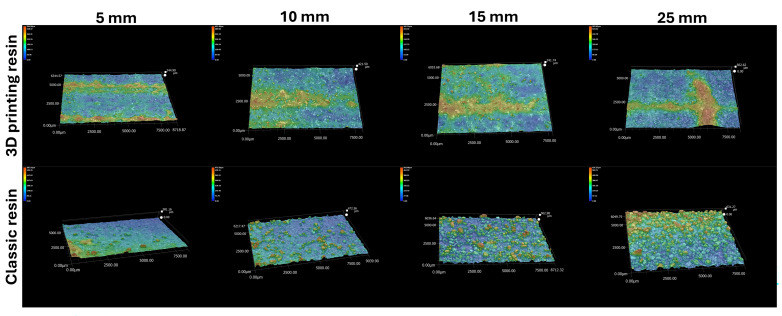
Roughness profiles of the tested castings depending on the type of resin used.

Figure 14 shows the roughness profiles of the tested castings depending on the wall thickness of the casting and the type of resin used.

**Figure 15:**
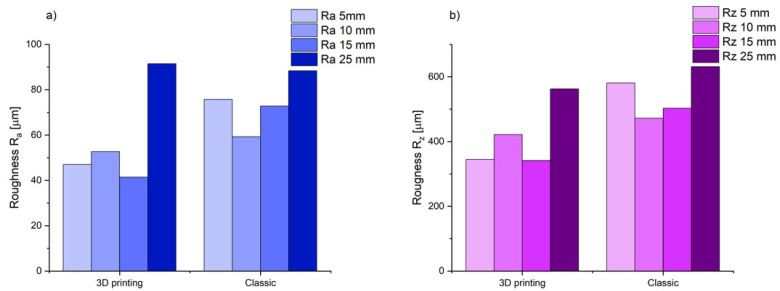
Summary of roughness results obtained from scanning the area using a Keyence VHX7100 microscope: (**a**) arithmetic mean deviation of the profile from the mean line (R_a_); (**b**) roughness height according to 10 points (R_z_).

Figure 15 shows the results of the arithmetic mean deviation of the profile from the mean line (R_a_) and the roughness height according to 10 points (R_z_) for the tested casting depending on the resin type used.

Measurements of roughness profiles (Figure 13) and R_a_ and R_z_ parameter values (Figure 14) show that the type of resin used has a significant impact on the surface quality of castings. Castings made using a classic binder show higher values in both the arithmetic mean profile deviation (R_a_) and the 10-point roughness height (R_z_) compared to castings made using a 3D printing binder.

### 3.4. Gases Emission Results

**Figure 16:**
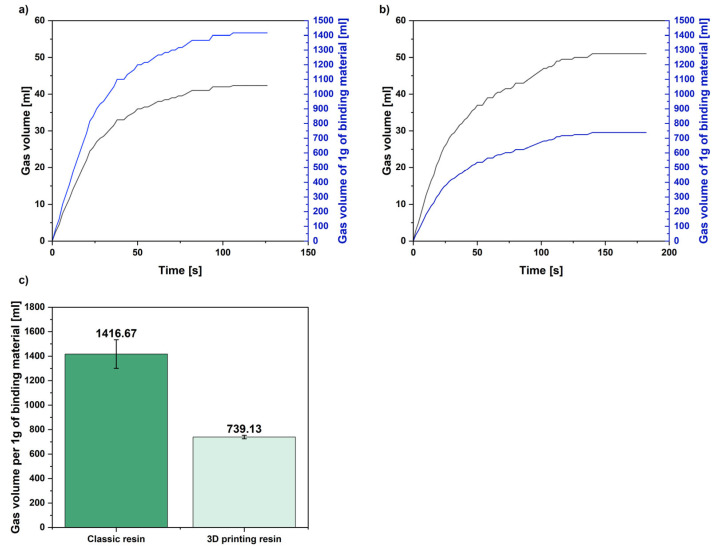
Results of gas emission tests with values calculated per 1g of binding material (binder + hardener), (**a**) classic resin, (**b**) 3D printing resin, (**c**) final value of emitted gases.

Figure 16 shows the results for the gas emissions of the tested molding sands.

The conducted research proved that molding sand with a binding system dedicated to 3D printing is characterized by app. 48% lower gas emissions calculated per 1 g of binding material compared with classic molding sand. This may be the reason for the higher surface roughness of castings produced in classic molds in comparison to castings produced in printed sand molds.

## 4. Discussion

The aim of this research was to determine the effect of different types of furfuryl resin on casting properties, such as surface quality and microstructure. Gas emission tests for molding sands with both types of resins were also conducted and analyzed together with roughness tests.

On the basis of the obtained research results, the following hypotheses were formulated and proved: H1: Resin type affects R_a_ and R_z_. H2: Resin type does not affect SDAS and L_max_. H3: Reduction in gassing correlates with a reduction in R_z_.

As part of this research, a casting mold was designed, for which a simulation of the pouring and solidification process was carried out. Analysis of the results allowed us to determine that the casting mold and the feeding system were designed correctly, ensuring the smooth flow of liquid metal through the pouring channels. At the same time, the mold design ensures a stable alloy temperature during the pouring process. The mold heats up evenly, with slight temperature increases occurring at the heat nodes. It was shown that the probability of shrinkage porosity is low, which confirms the correct feeding of the casting during the solidification process.

A gray cast iron casting was tested. Microstructure tests were performed to determine the effect of the casting wall thickness and the resin used on the structure of the casting surface layer. Tests on non-etched samples showed that the microstructure is dominated by visible graphite flakes, characteristic of gray cast iron. A pearlite structure with possible ferrite bands is visible, which also confirms the typical microstructure of gray cast iron. In the case of thinner walls (5 mm and 10 mm), a clearly finer structure is observed, which is the result of faster heat removal during solidification. In the case of samples with a thickness of 15 mm and 25 mm, the structure is coarser, which indicates slower cooling and longer crystallization time. This is a typical phenomenon for castings with different wall thicknesses. In the case of etched samples, the matrix is more clearly visible, making it easier to assess the homogeneity of the structure and potential casting defects. When comparing the effect of the type of molding sand—with resin intended for 3D printing and classic resin—no significant differences in the shape and distribution of graphite are observed. The research, therefore, shows that the type of resin used as a binder does not affect the microstructure of the casting, which proves research hypothesis H2.

Our analysis of three-dimensional roughness profiles allowed for a comparison of the impact of the resin used on the surface quality of the castings. The results of the tests clearly showed differences in the surface topography of the castings examined, which proved research hypothesis H1. It was observed that the roughness parameters are lower in the case of castings made in a mold with resin for 3D printing. The casting’s surface is smoother with a more uniform structure. In the case of a casting made in a mold with classic resin, greater variability in surface topography was observed, with clear traces of shrinkage, which is the result of liquid metal penetrating deep into the mold. As the wall thickness of the casting increases, its surface roughness increases (an increase in R_a_ and R_z_ parameters). This is due to the longer solidification and crystallization time (as shown by numerical simulations), and thus, the liquid casting alloy may penetrate deeper layers of the mold.

The increased roughness of the castings produced in molds made from classic furfuryl resin may also be caused by a higher amount of gas emitted from this molding compound, as demonstrated by gas emission tests. This proved research hypothesis H3. The gases released have a significant impact on the roughness of castings. With higher gas generation, the gases released as a result of the binding material burning out in the top layers of the casting mold can cause open blisters to form. Also, after the binder burns out, unbound sand can stick to the surface, which, after initial cleaning of the raw surface layer of the casting, can also increase the roughness of the top layer of the finished part [28].

Based on the results of these tests, it can be concluded that in the production of cast iron castings, it is more advantageous to use 3D-printed molds than those made using classical technology. The surface of the castings is better, and the amount of gas emitted is lower. However, it is currently not possible to replace the classic technology of mold and core production with additive technology. There are limitations related to, for example, the size of printing devices and production time. However, over time, additive technologies will likely partially replace the production of casting molds intended for the production of iron alloy castings.

The aim of the research presented in this article was to determine the differences in the amount of gas emitted by classic molds and molds printed using 3D technology during the production of cast iron castings. Future research will include analyzing the composition of the emitted gases and their impact on the environment. When using organic binders, lower gas emissions are beneficial for both environmental and technological reasons.

## 5. Conclusions

The conducted research on the influence of furfuryl resin type on casting properties allowed for the formulation of the following conclusions:

- Numerical simulations confirmed the correctness of the design of the pouring system and the casting mold, allowing for uniform heating and smooth metal flow in the mold.
- The thickness of the casting wall affects both the microstructure and the surface roughness, while the type of resin does not significantly affect the matrix (perlite/ferrite), SDAS, or L_max_; a difference in N_A_ is observed.
- The type of resin used affects the surface quality of castings, as was confirmed by surface topography images and roughness parameter results, R_a_ and R_z_.
- Gas emission tests have shown that a greater amount of gas is emitted from molds made using conventional technology. This affects the surface quality of the produced casting.
- The next research step would be the elaboration of molding materials for the 3D printing of molds with the use of inorganic binders, including the possibility of using aluminosilicates. We also plan to test the mechanical properties and corrosion resistance of cast iron castings in future research.

## Figures and Tables

**Table 1:** Compositions of molding sands used for tests.

Molding Sand	Molding Sand’s Composition
Classic	Quartz sand, 100 p.p.w. Classic furfuryl resin, 1.0 p.p.w. Classic hardener, 0.5 p.p.w.
For 3D printing	Quartz sand, 100 p.p.w. Furfuryl resin designed for 3D printing, 1.0 p.p.w. Hardener designed for 3D printing, 0.5 p.p.w.

**Table 2:** Main resin properties.

Feature	Classic Furfuryl Resin	Furfuryl Resin Designed for 3D Printing
Density (20 °C), g/cm^3^	1.175–1.185	1.1–1.2
Viscosity (20 °C), mPa·s	30–45	2–6
Furfuryl alcohol, %	75 ± 1	70–90
Free formaldehyde, %	0.12–0.14	-
Nitrogen, %	3.5 ± 0.5	-

**Table 3:** Main hardener properties.

Feature	Classic Furfuryl Resin	Furfuryl Resin Designed for 3D Printing
Density (20 °C), g/cm^3^	1.225 ± 0.01	1.2–1.3
Viscosity (20 °C), mPa·s	15	10–30
Acid content, g/g of KOH	0.225 ± 0.01	-
Color	Yellowish, clear	Yellowish-brown, clear

**Table 4:** Chemical composition analysis of the examined cast iron.

Element	Content, % wt.
C	3.26
Si	1.57
Mn	0.65
P	0.03
S	0.04
Fe	Balance

**Table 5:** Metallography analysis of the examined cast iron castings.

Resin	Wall Thickness, mm	Type of Graphite, %	L_max_, mm	SDAS, mm	N_A_, cm^−2^	Ferrite Fraction, %
Classic resin	5	100%D	28.5	22.8	2388	~3
	10	20%B 30%D 50%E	60.1	26.3	1846	~3
	15	10%A 20%D 70%E	127.5	40.2	503	~3
	25	40%A 30%D 30%E	192.2	49.1	334	~3
3D printing resin	5	100%D	43.5	18.6	1490	~3
	10	10%B 90%D	74.5	27.9	1208	~3
	15	10%E 10%D 10%B 70%A	244.8	45.8	336	~3
	25	30%B 20%D 20%E 30%A	223.4	54.6	312	~3

Type of graphite: A—uniformly distributed, B—rosette-like, D—fine–interdendritic, E—coarse–interdendritic, according to EN ISO 945-1:2008. L_max_—maximum length of graphite flake particles, µm; SDAS—secondary dendrite arm spacing, µm; N_A_—eutectic grains per unit area, cm^−2^.

## Data Availability

The original contributions presented in this study are included in the article. Further inquiries can be directed to the corresponding authors.
